# Neutralizing Activity of SARS-CoV-2 Antibodies in Patients with COVID-19 and Vaccinated Individuals

**DOI:** 10.3390/antib12040061

**Published:** 2023-09-25

**Authors:** Tatjana Vilibic-Cavlek, Vladimir Stevanovic, Snjezana Kovac, Ema Borko, Maja Bogdanic, Gorana Miletic, Zeljka Hruskar, Thomas Ferenc, Ivona Coric, Mateja Vujica Ferenc, Ljiljana Milasincic, Ljiljana Antolasic, Ljubo Barbic

**Affiliations:** 1 Department of Virology, Croatian Institute of Public Health, 10000 Zagreb, Croatia; emaborko01@gmail.com (E.B.); maja.bogdanic@hzjz.hr (M.B.); zeljka.hruskar@hzjz.hr (Z.H.); ljiljana.milasincic@hzjz.hr (L.M.); ljiljana.antolasic@hzjz.hr (L.A.); 2 School of Medicine, University of Zagreb, 10000 Zagreb, Croatia; 3 Department of Microbiology and Infectious Diseases with Clinic, Faculty of Veterinary Medicine, University of Zagreb, 10000 Zagreb, Croatia; skovac@vef.hr (S.K.); gmiletic@vef.hr (G.M.); icoric@vef.hr (I.C.); ljubo.barbic@vef.hr (L.B.); 4 Clinical Department of Diagnostic and Interventional Radiology, Merkur University Hospital, 10000 Zagreb, Croatia; thomas.ferenc95@gmail.com; 5 Department of Obstetrics and Gynecology, University Hospital Center Zagreb, 10000 Zagreb, Croatia; matejavujica1@gmail.com

**Keywords:** COVID-19, SARS-CoV-2, variants of concern, EIA, virus neutralization test, cross-neutralization

## Abstract

Background: Serological diagnosis of COVID-19 is complex due to the emergence of different SARS-CoV-2 variants. Methods: 164 serum samples from (I) patients who recovered from COVID-19 (*n* = 62) as well as (II) vaccinated individuals (*n* = 52) and (III) vaccinated individuals who were infected with different SARS-CoV-2 variants after vaccination (*n* = 50) were included. All samples were tested using EIA (binding antibodies) and a virus neutralization test (VNT) using the Wuhan strain (NT antibodies). Group III was further tested with a VNT using the Alpha/Delta/Omicron strains. Results: The highest antibody index (AI) was observed in vaccinated individuals infected with COVID-19 (median AI = 50, IQR = 27–71) and the lowest in vaccinated individuals (median AI = 19, IQR = 8–48). Similarly, NT antibody titer was highest in vaccinated individuals infected with COVID-19 (median 128; IQR = 32–256) compared to vaccinated individuals (median 32, IQR = 4–128) and patients with COVID-19 (median 32, IQR = 8–64). The correlation between AI and NT titer was strongly positive in vaccinated individuals and moderately positive in patients with COVID-19. No significant correlation was observed in vaccinated individuals infected with COVID-19. In patients infected with Alpha and Delta, the lowest VNT positivity rate was for the Omicron variant (85.0%/83.3%). Patients infected with the Alpha variant showed the lowest NT titer for the Omicron variant (median titer 32) compared to the Wuhan/Delta variants (64/128). Patients infected with the Delta variant had the lowest NT titer to the Omicron variant (median 32), compared to the Wuhan/Alpha variants (64/128). Patients infected with the Omicron variant showed similar titers to the Delta/Wuhan variants (128) and higher to the Alpha variant (256). Conclusions: The cross-immunity to SARS-CoV-2 is lowest for the Omicron variant compared to the Alpha/Delta variants.

## 1. Introduction

Since the first cases of coronavirus disease (COVID-19) were recorded in Wuhan, China, in 2019, the world has faced a global pandemic, with more than 768 million cases, and nearly 7 million deaths due to the severe acute respiratory syndrome coronavirus 2 (SARS-CoV-2) [1]. Like other coronaviruses, SARS-CoV-2 has a positive-sense single-stranded RNA genome that encodes four structural proteins, including spike (S), envelope (E), membrane (M), and nucleocapsid (N), as well as sixteen non-structural proteins (nsp1−16). The S protein consists of two functional subunits, S1 and S2. The S1 subunit contains a receptor-binding domain (RBD) that recognizes and attaches to the host receptor (angiotensin-converting enzyme 2; ACE-2), whereas the S2 subunit mediates the fusion of the virus and host cell membranes [2]. While the S protein is an important target for inducing neutralizing (NT) antibodies, the antibodies to the N protein are less likely to neutralize the virus [3].

Studies on the SARS-CoV-2 genome have shown mutations that could change a virus’s virulence and transmissibility. Mutations in the genome occur due to errors during RNA replication, resulting in many SARS-CoV-2 variants [4]. The high genetic recombination and mutation rates of SARS-CoV-2 contribute to its ecological diversity [5]. Two important types of variants are of public health interest: variants of concern (VOCs) and variants of interest (VOIs). Due to mutations in the S protein, a number of VOCs have emerged including Alpha (B.1.1.7), Beta (B.1.351), Gamma (P.1), Delta (B.1.617.2), and Omicron (B.1.1.529), which are the major SARS-CoV-2 VOCs [6,7,8,9]. SARS-CoV-2 mutations affect the properties of the virus, including cell tropism, virus transmissibility, antigenicity, and a significant decrease in NT activity induced by prior infection or vaccination [10].

The Alpha variant, first detected in the United Kingdom in September 2020, possesses a wide range of mutations, some of which have greatly affected the function of the S protein [4,11]. In addition, a 50–100% higher reproduction number compared to other non-VOC lineages was observed [12]. The Beta variant appeared in South Africa in September 2020. According to the epidemiological data, the percentage of Beta variant-associated infections increased to ∼50% of the total daily infections within approximately three months since its outbreak [13]. Beta variants contain nine S mutations, of which the most significant substitutions of the amino acids are located in the RBD region [4]. Because mutations in the Beta variant cause escape from NT antibodies, the possibility for reinfection is relatively high [14]. Vaccines also appear less effective in preventing COVID-19 caused by the Beta variant. The Gamma variant, first detected in Brazil (September 2020), possesses some of the same S protein mutations as the Alpha and Beta variants, which promote attachment to human cells [11,15]. The Gamma variant caused a higher viral load and showed 1.4–2.2 times more transmissibility than previous SARS-CoV-2 variants [14]. The Delta variant occurred in March 2021 in India [11]. Studies have shown that the Delta variant is 60–80% more transmissible than the Alpha variant [13,15]. Two mutations (L452R and E484Q) in the S protein of the Delta variant are particularly significant for SARS-CoV-2 infectivity, which may reduce the antibody effectiveness from previous viral variants and vaccination [4]. Vaccines seem less effective against the Delta than the Alpha variant, but they are still highly effective in COVID-19 prevention after two vaccine doses [15]. After the first detection of the Omicron variant (September 2021, South Africa and Botswana), this variant spread and rapidly replaced the Delta variant. In contrast to the Delta variant with two mutations, 15 mutations have been detected in the RBD of the Omicron variant, the major target of the NT antibodies. Numerous non-synonymous mutations were observed in the S protein, some of which have been linked to higher binding affinity to the ACE-2 receptor, enhanced transmissibility, reduced ability of neutralization by antibodies, and immune escape. More than 60 mutations have been detected in Omicron, making it a variant with the largest number of mutation sites of all SARS-CoV-2 variants [13].

Several commercial tests for SARS-CoV-2 serological diagnosis are available, such as rapid immuno-chromatographic tests (ICTs), enzyme immunoassays (EIAs), indirect immunofluorescence assays (IFAs), and chemiluminescence assays (CLIAs) [16]. These detect antibodies to the virus RBD, the entire S and N antigen, or all three [17]. The EIA is the most widely used serological screening test that detects SARS-CoV-2 binding antibodies; however, it cannot provide information on the effectiveness of functional antibodies, which correlate with protection against infection [18]. In addition, because of the potential cross-reactive properties of binding antibodies with seasonal coronaviruses, the virus neutralization test (VNT) using cell culture remains the “gold standard” test for the serological diagnosis of SARS-CoV-2 infection [17].

The immune response to COVID-19 has become complex and heterogeneous due to the emergence of different SARS-CoV-2 variants, especially Omicron and its subvariants [19]. Compared to the SARS-CoV-2 Wuhan strain, a high number of mutations in the S protein of the Omicron variant, especially in the RBD, were detected [20]. Reduced serum NT antibody activity against some SARS-CoV-2 VOCs has been observed in convalescents or vaccinated individuals who received various types of COVID-19 vaccines, increasing the risk of SARS-CoV-2 reinfection or post-vaccination infection [21,22].

Vaccination against SARS-CoV-2 only induces the production of S-protein-targeting antibodies, unlike natural infection, which results in the development of antibodies against the RBD and the S1, but also against the S2 as well as the N domain [23]. While antibodies to the N protein are likely non-NT, antibodies to the N domain of S1 (outside of the RBD) have shown NT potential [24]. The SARS-CoV-2 S2 domain-targeting antibodies also exhibit NT properties [25].

Like the rest of the world, several SARS-CoV-2 epidemic waves driven by different variants were observed in Croatia. Virus lineages belonging to clades G, GR, and GV dominated during the first and second epidemic waves. A noticeable weekly increase in the Alpha variant indicated the start of the third epidemic wave. Beginning in March 2021, the Alpha variant predominated (more than 50% of positive samples). The first Delta variants were observed in the first week of June 2021, rapidly replacing the Alpha variant and reaching more than 90% of all sequenced samples by the beginning of August 2021. Beta and Gamma variants were documented in a small number of samples. The first case of the Omicron variant was detected in November 2021 and spread rapidly, causing the fifth epidemic wave [26,27,28].

The aim of this study was to evaluate the NT activity of SARS-CoV-2 antibodies in patients who recovered from COVID-19, SARS-CoV-2 vaccinated individuals, and patients with “hybrid immunity” (vaccinated individuals infected with different SARS-CoV-2 VOCs after vaccination).

## 2. Materials and Methods

### 2.1. Patients

**Figure 1:**
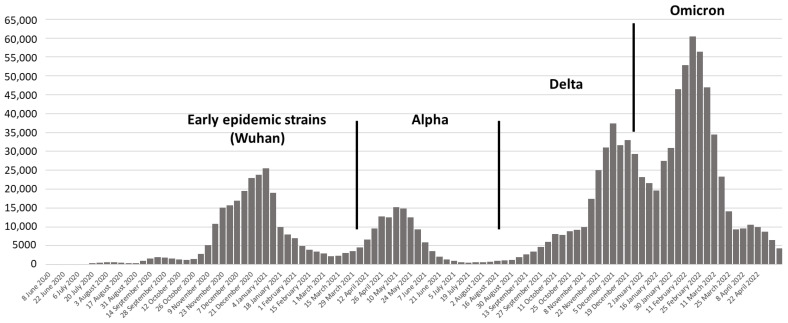
Dominant SARS-CoV-2 variants in COVID-19 epidemic waves in Croatia.

A total of 164 serum samples from patients recovered from COVID-19 and/or vaccinated individuals collected during a two-year period (May 2020–April 2022) were included in the study. The patients were divided into three groups: (I) patients who recovered from COVID-19 during the first epidemic wave (*n* = 62); (II) SARS-CoV-2 vaccinated individuals (*n* = 52); and (III) vaccinated individuals who were infected with SARS-CoV-2 after vaccination during the third, fourth, and fifth epidemic waves (*n* = 50) (Figure 1).

Vaccinated individuals received two doses of mRNA COVID-19 vaccines (BNT162b2; Pfizer or mRNA-1273; Moderna) or one dose of the adenovirus vector vaccine (Ad26.COV2.S; Johnson and Johnson), designed using the S gene sequence of the original SARS-CoV-2 strain [29,30,31]. Patients who were SARS-CoV-2 infected after vaccination were further subdivided according to the variant: Alpha (*n* = 20), Delta (*n* = 18), and Omicron (*n* = 12).

**Figure 2:**
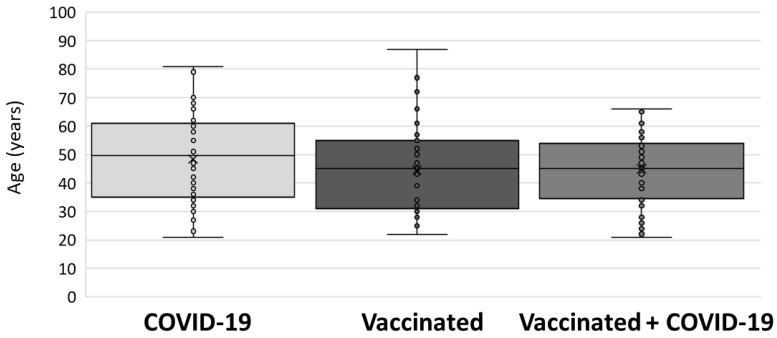
Age of participants included in the study.

The age of study participants in each tested group is presented in Figure 2. No significant difference between groups (*p* = 0.276) was observed. Median age (interquartile range: IQR) was as follows: 49 (35–61) years (group I); 45 (31–56) years (group II); and 45 (34–54) years (group III).

### 2.2. Methods

All samples were initially tested using EIA (SARS-CoV-2 binding antibodies) and VNT (early-epidemic Wuhan strains, NT antibodies). Samples from vaccinated individuals infected with COVID-19 were additionally tested for the presence of NT antibodies against Alpha, Delta, and Omicron SARS-CoV-2 strains.

Initial serological screening (detection of binding antibodies) was performed using a commercial indirect EIA using recombinant S and N antigens of SARS-CoV-2 (ELISA COVID-19 IgG; Vircell Microbiologists, Granada, Spain). The EIA method is based on the reaction of antibodies present in a serum sample with antigens immobilized on a solid phase (microtiter plate). After washing off unbound immunoglobulins, peroxidase-labeled anti-human IgG conjugate binds to the antigen–antibody complex in a second step. After another washing step, tetramethylbenzidine substrate is added, producing a colored soluble product. Finally, 0.5 M sulfuric acid is added to stop the reaction. The optical densities are measured using a spectrophotometer at a wavelength of 450 nm and a reference wavelength of 620 nm. The antibody index (AI) was calculated. AI = (sample OD/mean cut-off serum OD) × 10 and interpreted as follows: IgG AI < 4, negative; 4–6, borderline; >6, positive [32].

NT antibodies were detected using a VNT in cell culture. The virus isolates used in this study were obtained from nasopharyngeal swabs of RT-PCR-positive Croatian patients. The SARS-CoV-2 Wuhan, Alpha, Delta, and Omicron strains isolated in Vero E6 cell culture (ATCC CRL-1586) were used as a stock virus. The Reed and Muench formula was used to calculate the virus titer (50% tissue culture infectious dose: TCID_50_) [33]. Two-fold serial dilutions of heat-inactivated serum samples (30 min at 56 °C) were prepared (starting with 1:2). A mixture of an equal volume (25 μL) of diluted inactivated serum samples and 100 TCID_50_ of SARS-CoV-2 was incubated at 37 °C with CO_2_ for one hour. In the final step, 50 μL of 2 × 10^5^ Vero E6 cells/mL were added to each well. To ensure optimal test results, the virus antigens used in each run were back-titrated, and a positive control sample of known titer and a negative control sample were included in each microtiter plate. The plates were incubated at 37 °C with CO_2,_ and from the third day, the plates were examined for the cytopathic effect. The antibody titer was defined as the reciprocal of the highest serum dilution showing at least 50% neutralization. NT antibody titer ≥8 was considered positive.

### 2.3. Statistical Analysis

The SARS-CoV-2 positive detection rates were presented as numbers and percentages with 95% confidence intervals (CI). The Chi-square test was used to compare the differences in the NT antibody positive detection rates between groups. The correlation between EIA and VNT was calculated using Spearman’s rank correlation coefficient. A Kruskal–Wallis test was used to compare the differences in the NT antibody titers. A *p*-value < 0.05 was considered statistically significant. The Social Science Statistics program (https://www.socscistatistics.com/tests/ (accessed on 10 August 2023) was used for statistical analysis.

## 3. Results

**Figure 3:**
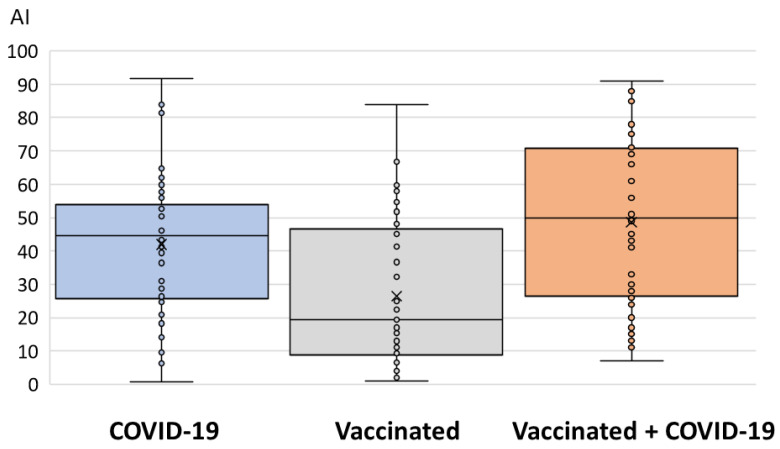
Binding antibody levels (EIA) in three groups of patients.

**Figure 4:**
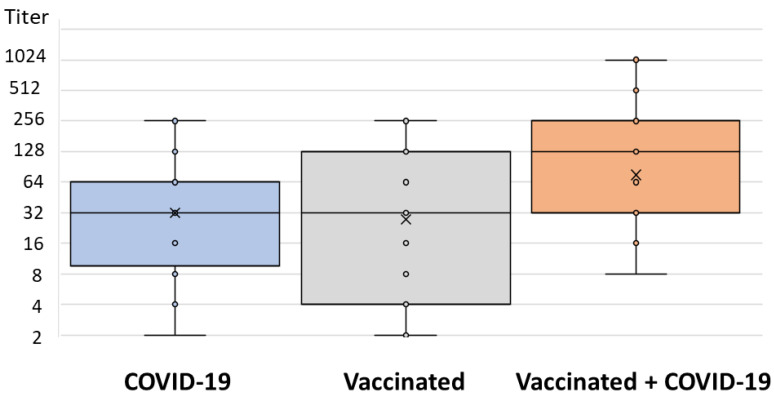
Neutralizing activity (VNT, Wuhan strain) in three groups of patients.

The median antibody levels detected via EIA (binding antibodies) and VNT (NT antibodies against the Wuhan strain) are presented in Figure 3 and Figure 4. Levels of binding antibodies differed significantly between groups (*p* < 0.001). A high AI was observed in vaccinated individuals infected with COVID-19 (median AI = 50, IQR = 27–71) and patients with COVID-19 (median AI = 44.5, IQR = 26–54), while it was lower in vaccinated individuals (median AI = 19, IQR = 8–48) (Figure 3).

In addition, significant differences were observed in NT antibody titers between groups (*p* = 0.003): vaccinated individuals infected with COVID-19 exhibited a median NT titer of 128 (IQR = 32–256), patients with COVID-19 exhibited a median titer of 32 (IQR = 8–64), and vaccinated individuals exhibited a median titer 32 (IQR = 4–128) (Figure 4).

**Figure 5:**
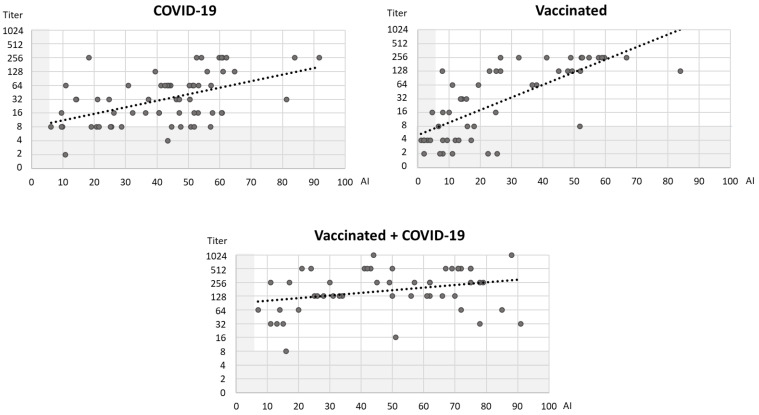
Correlation of AI (EIA) and NT titer (VNT, Wuhan) in three groups of patients (gray shadowed areas represent cut-off values for EIA and VNT; black dotted lines represent trendlines).

Comparing the EIA (AI) and VNT (NT titer) results, the correlation between AI and NT titer was strongly positive in vaccinated individuals (Spearman’s rho = 0.763; *p* < 0.001) and moderately positive in patients with COVID-19 (Spearman’s rho = 0.468; *p* < 0.001). No significant correlation was observed in vaccinated individuals infected with COVID-19 (Spearman’s rho = 0.253; *p* = 0.075) (Figure 5).

The NT activity of SARS-CoV-2 antibodies to different viral strains in vaccinated individuals infected with COVID-19 according to the infection strain is presented in Table 1. In patients infected with the Alpha strain, NT antibody positivity rates for the Delta, Omicron, and Wuhan strains were 100, 85.0, and 90.0%, respectively (*p* = 0.217). In patients infected with the Delta strain, significant differences in the SARS-CoV-2 NT activity were observed. The lowest NT positivity was detected for the Omicron strain (83.3%), while all samples neutralized the Alpha and Wuhan strains (*p* = 0.041). In patients infected with the Omicron strain, the positive detection rates were 100% for Alpha and Delta and 91.7% for the Wuhan strain (*p* = 0.357).

**Figure 6:**
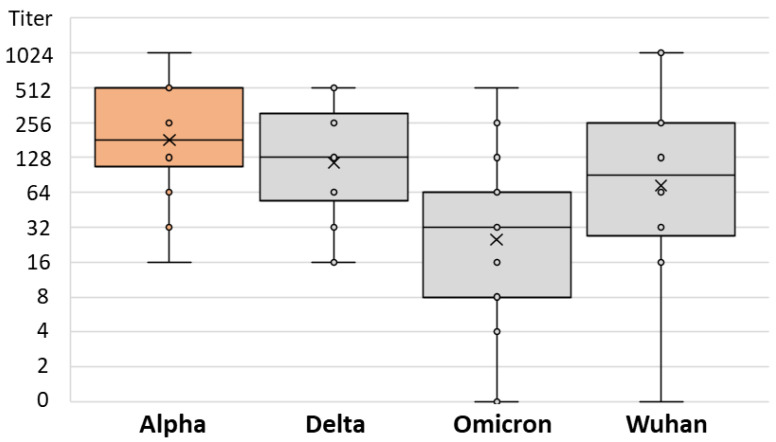
SARS-CoV-2 neutralizing antibody titers in vaccinated individuals infected by Alpha variant.

**Figure 7:**
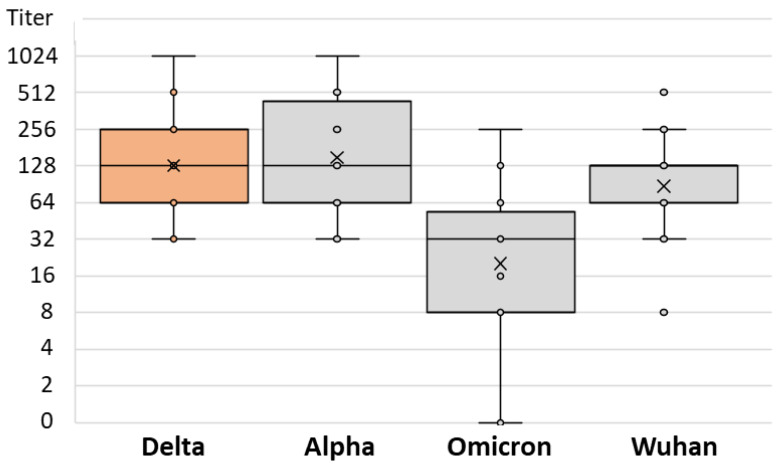
SARS-CoV-2 neutralizing antibody titers in vaccinated individuals infected by Delta variant.

**Figure 8:**
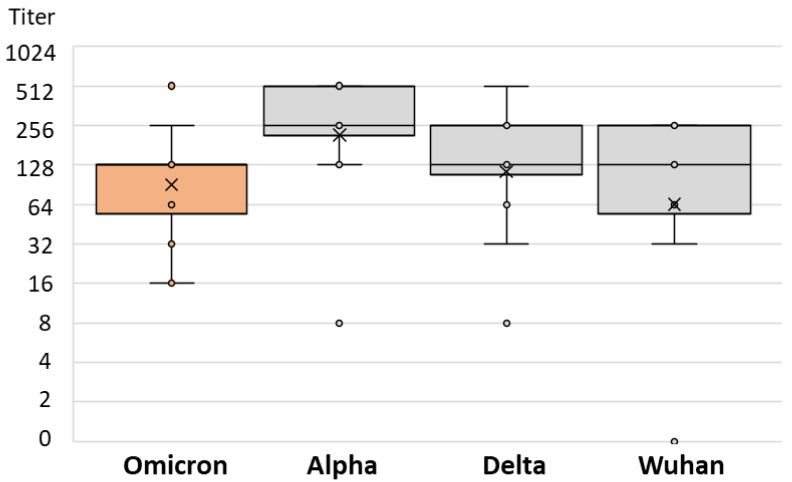
SARS-CoV-2 neutralizing antibody titers in vaccinated individuals infected by Omicron variant.

There was no difference in the SARS-CoV-2 NT antibody-specific titers in patients infected after vaccination with the Alpha, Delta, and Omicron strains (median titers 256, 128, and 128, respectively; *p* = 0.234) (Table 2). However, the levels of NT antibody titers to different SARS-CoV-2 strains differed significantly among patients infected with different SARS-CoV-2 variants. Patients infected with the Alpha variant showed a lower NT titer for the Omicron variant (median titer 32) than for the Wuhan and Delta strains (64 and 128, respectively; *p* = 0.013; Table 2 and Figure 6). Similarly, patients infected with the Delta variant had the lowest NT antibody titer to the Omicron variant (median 32), while it was higher for the Wuhan and Alpha variants (64 and 128, respectively; *p* = 0.001; Table 2 and Figure 7). Patients infected with the Omicron variant showed a similar NT titer (128) to Delta and Wuhan, while it was higher for the Alpha variant (256; *p* = 0.029; Table 2 and Figure 8).

## 4. Discussion

In the presented study, two serological methods (EIA and VNT) for SARS-CoV-2 diagnosis were analyzed in three groups of patients. The highest levels of binding antibodies (EIA) were detected in patients with “hybrid immunity” (vaccinated individuals who developed SARS-CoV-2 infection after vaccination; median AI: 50), followed by patients with COVID-19 (median AI: 44.5) and vaccinated individuals (median AI: 19). NT antibody titers were also significantly higher in vaccinated individuals infected with COVID-19 (median titer: 128) compared to COVID-19 patients and vaccinated individuals (median titer: 32 each). It was shown that SARS-CoV-2 NT titers increased as a result of immunization or booster vaccinations combined with repeated infection-related immunizing exposures [34].

Similar to a previous Croatian study [32], a significant positive correlation was observed between EIA and VNT (original Wuhan strain) in patients who were infected with COVID-19 and vaccinated individuals, while there was no significant correlation in vaccinated individuals infected with COVID-19.

Understanding the degree of protection against new SARS-CoV-2 variants with immune escape properties after both vaccination and natural infection is important during the ongoing COVID-19 epidemic. Some studies demonstrated that patients infected with wild-type (Wuhan) SARS-CoV-2 strains possess cross-NT antibodies which play an important role in polyclonal neutralization for some of the VOCs. Changrob et al. studied samples collected from individuals naturally infected with the Wuhan strain. Twelve NT monoclonal antibodies against three distinct regions on the S protein that are able to induce the neutralization of the Alpha, Gamma, and Delta SARS-CoV-2 variants were identified. However, the convalescent participants only had adequate antibody titers to neutralize the Alpha and Gamma variants, not the Delta variant [35].

Several studies have demonstrated that prior SARS-CoV-2 infection can significantly enhance the antibody response to COVID-19 vaccination [36,37], however; little is known about how infection in vaccinated individuals will affect the magnitude and extent of the NT antibody response.

In our study, in patients with the “hybrid immunity” and post-vaccinal SARS-CoV-2 infection caused by the Alpha and Delta variants, the lowest cross-neutralization rate was observed with the Omicron variant (85.0 and 83.3%, respectively). All patients infected with the Alpha variant cross-neutralized the Delta strain, and 90.0% neutralized the Wuhan strain. In patients infected with the Delta variant, the percentage of cross-neutralization with both Alpha and Wuhan was 100%.

Some studies have shown that vaccinated individuals developed high NT antibody titers against all VOCs after breakthrough Omicron infections. However, primary Omicron infection causes only limited cross-variant neutralization in unvaccinated people. Therefore, Omicron infection can enhance vaccination-induced existing immunity but may not protect non-vaccinated individuals from reinfection by other VOCs [38,39]. Similar to these observations, our results showed that all individuals (100%) infected with the Omicron strain after vaccination neutralized the Alpha and Delta variants, while the neutralization rate of the Wuhan strain was 91.7%.

A study from the USA analyzed the NT antibody responses and the cross-NT immunity in patients with confirmed SARS-CoV-2 breakthrough infections. Similar to our results, the study results suggest that Omicron breakthrough infections are less immunogenic than Delta infections, resulting in less protection against reinfection or infection with new SARS-CoV-2 variants [40]. In addition to a high number of nonsynonymous mutations in the S protein [41], a possible reason may be an increased proportion of asymptomatic or mild infections caused by the Omicron variant compared to other viral variants [42].

A German study analyzed the serum NT activity against the Wuhan, Alpha, Beta, Delta, and Omicron SARS-CoV-2 strains after the second dose of the BNT162b2 vaccine. A significantly higher serum NT activity against all investigated variants was observed in vaccinated individuals with subsequent non-Omicron SARS-CoV-2 infection compared to individuals who received two vaccine doses and experienced no subsequent infection [43]. These results are in line with the results presented in our study.

An Italian study investigated the interaction of COVID-19 immunity after natural infection and vaccination in inducing protective immunity in a cohort of patients who were infected with SARS-CoV-2 during the first epidemic wave (February 2020). The tested groups included unvaccinated previously infected individuals, vaccinated–naïve, and vaccinated–previously infected individuals. Compared to the NT serum activity against the original ancestral B.1 strain, the reactivity against B.1.617.2 (Delta) and Omicron BA.1 was 4-fold and 16-fold lower. A statistically significant reduction in antibody titers against the S protein among unvaccinated individuals and an increase in vaccinated individuals was observed, whereas antibody titers against N antigens decreased in both groups. Furthermore, compared to the control group, vaccinated individuals previously infected with SARS-CoV-2 induced higher antibody levels than non-infected but vaccinated participants. Authors also identified a more robust immune response against the S protein following two doses of a vaccine compared to naturally infected subjects, however, still with reduced neutralization of the Delta and Omicron variants [44].

Furthermore, a study from Brazil showed that prior infection with the Wuhan strain correlated with positive NT antibodies against the Wuhan, Gamma, and Omicron variants in 93.3, 77.6, and 1.7% of serum samples, respectively [45].

A Korean study evaluated the duration of antibody response and cross-NT activity against the Alpha, Beta, and Delta variants following infection by the Wuhan variant. In the first 11 months after COVID-19 diagnosis, the levels of NT antibodies against the Wuhan variant remained positive in most patients with a gradual decrease after 4, 6, and 11 months (NT antibodies present in 98.5, 86.8, and 58.8% of patients, respectively). Using the Wuhan variant as a comparison, NT titers against the Alpha variant did not differ significantly; however, weakened cross-neutralization against the Beta and Delta variants was observed [46].

Analyzing the cross-reactive NT titers in our study, the levels of NT antibodies to different SARS-CoV-2 variants differed significantly among patients infected with different viral strains. After vaccination, patients infected with the Alpha and Delta variants showed the lowest NT titers to the Omicron variant (median titer 32).

A Canadian study also analyzed the NT potential of serum samples from patients recovering from COVID-19 and individuals vaccinated against ancestral SARS-CoV-2 (Wuhan) and VOCs during the first wave. Convalescent sera from COVID-19 patients infected with the ancestral strain demonstrated lower NT titer against Beta and Omicron VOCs but were not significantly different between ancestral SARS-CoV-2 and the Delta variant. In contrast, samples from individuals who were naturally infected with the ancestral SARS-CoV-2 and subsequently received two BNT162b2 vaccine doses developed significantly higher NT antibody levels against the ancestral virus as well as all VOCs. Convalescent samples from patients infected with the Delta variant contained lower NT antibody levels against both Beta and Omicron variants, whereas titers against ancestral SARS-CoV-2 and Delta variants were similar [47]. A great number of subvariants and mutations on the S protein allow the Omicron variant to successfully evade immunity from NT antibodies that are produced either through natural infection or vaccination [9,45].

Some limitations of this study need to be addressed, such as the small sample size. In addition, data on the time of the vaccination were not available for the majority of patients; therefore, sample collection dates after vaccination may differ, which could have at least partly impacted the serology results.

Numerous studies have indicated that cohorts of people with chronic diseases and those receiving immunosuppressive medication have inadequate immune responses to COVID-19 [48]. The hemodialysis population does not show the same serological response to SARS-CoV-2 vaccination as immunocompetent people [49]. In addition, the serological response to SARS-CoV-2 vaccination is also diminished in the elderly. After immunization, there is a trend of declining humoral response, which is especially pronounced in people over 65 years of age [50].

Immunocompromised patients showed altered SARS-CoV-2 antibody kinetics, i.e., delayed immunologic response. A study conducted in Massachusetts, USA, measured the NT responses against the ancestral and major variants in lung and heart transplant recipients uninfected with SARS-CoV-2, cystic fibrosis patients, and healthy controls. The healthy control group showed a strong anti-S response immediately after vaccination, with cross-neutralization of all variants. The antibody titers increased gradually after the second vaccine dose in transplant recipients and showed no cross-neutralization of the Omicron B.1.1.529 variant. However, the majority of them displayed an improved but attenuated neutralization of the Beta, Gamma, and Omicron variants after the third dose [51].

Immunomodulatory therapy may induce a blunted humoral response to SARS-CoV-2 infection and reduce the response to vaccination [52,53].

Data on the immunosuppressive or immunomodulatory therapies for patients included in this study were not available, which is also a limiting factor. Therefore, it was not possible to analyze the potential impact of immunosuppression on the SARS-CoV-2 serological response.

## 5. Conclusions

The presented results showed significant variations in antibody levels with the highest levels (both in EIA and VNT) in patients with “hybrid immunity”. Through analyzing the NT activity in patients infected with different SARS-CoV-2 variants, the cross-immunity (frequency of antibody detection and NT antibody titers) to SARS-CoV-2 was found to be lower for the Omicron variant compared to the Alpha and Delta variants.

## Figures and Tables

**Table 1:** Neutralizing activity of SARS-CoV-2 antibodies in vaccinated individuals who developed COVID-19 after vaccination.

Postvaccinal Infection	SARS-CoV-2 Strain	NT Antibodies N (%)	95%CI	*p*
Alpha (*n* = 20)	Delta	20 (100)	83.1–100 *	
	Omicron	17 (85.0)	69.3–100	0.217
	Wuhan	18 (90.0)	76.8–100	
Delta (*n* = 18)	Alpha	18 (100)	81.5–100 *	
	Omicron	15 (83.3)	58.6–96.4	0.041
	Wuhan	18 (100)	81.5–100 *	
Omicron (*n* = 12)	Alpha	12 (100)	73.5–100 *	
	Delta	12 (100)	73.5–100 *	0.357
	Wuhan	11 (91.7)	61.5–99.8	

* One-sided 97.5% confidence interval.

**Table 2:** Neutralizing titers against different SARS-CoV-2 variants in vaccinated individuals who developed COVID-19 after vaccination.

Postvaccinal Infection	Infection NT Titer Range	Infection Median NT Titer (IQR)	SARS-CoV-2 Strain	Strain NT Titer Range	Strain Median NT Titer (IQR)	*p*
Alpha	16–1024	256 (128–512)	Delta	16–512	128 (64–256)	0.013
			Omicron	0–512	32 (8–64)	
			Wuhan	0–1024	64 (16–128)	
Delta	32–1024	128 (64–256)	Alpha	32–1024	128 (64–512)	0.001
			Omicron	0–256	32 (8–64)	
			Wuhan	8–1024	64 (32–128)	
Omicron	16–512	128 (64–128)	Alpha	8–512	256 (192–512)	0.029
			Delta	32–256	128 (128–128)	
			Wuhan	0–256	128 (96–256)	

## Data Availability

All related data and methods are presented in this paper. Additional inquiries should be addressed to the corresponding author.
